# Evaluation of a New Multiparametric Microdot Array-Based Immunoassay Panel for Systemic Autoimmune Disease Diagnosis

**DOI:** 10.3390/jpm14060607

**Published:** 2024-06-07

**Authors:** Maria Infantino, Francesca Pavia, Valentina Grossi, Barbara Lari, Maurizio Benucci, Francesca Li Gobbi, Silvia Pancani, Mariangela Manfredi

**Affiliations:** 1 Immunology and Allergology Laboratory, S. Giovanni di Dio Hospital, 50143 Florence, Italy; francesca.pavia@edu.unifi.it (F.P.); valentina2.grossi@uslcentro.toscana.it (V.G.); barbara.lari@uslcentro.toscana.it (B.L.); mariangela.manfredi@uslcentro.toscana.it (M.M.); 2 Rheumatology Unit, S. Giovanni di Dio Hospital, 50143 Florence, Italy; maurizio.benucci@uslcentro.toscana.it (M.B.); francesca.ligobbi@uslcentro.toscana.it (F.L.G.); 3 IRCCS Fondazione Don Carlo Gnocchi, 50143 Florence, Italy; silvia_pancani@hotmail.it

**Keywords:** microdot array, systemic autoimmune disease, new method, CTD screen

## Abstract

Background: The early reliable detection and quantification of autoantibodies play an important role in autoimmune disease diagnosis and in disease-course monitoring. New technologies, such as the multiplexed determination of autoantibodies, have recently been introduced and are being adopted more frequently. The aim of this study was to evaluate the ability of a new microdot array-based multiparametric assay (ZENIT AMiDot CTD panel, A. Menarini Diagnostics, Firenze, Italy) to correctly classify patients with autoimmune rheumatic diseases (ARDs) and compare it to a fluorescence enzyme immunoassay (FEIA) for the detection of anti-ENAs. Methods: The study included 69 consecutive samples from patients with ARDs that were analyzed using two different methods (FEIA and AMiDot) to detect anti-CENP B and six anti-ENA antibodies: anti-Scl-70, anti-SSB/La, anti-Jo-1, anti-U1-RNP, anti-Ro52, and anti-Ro60. The control group sera came from sixty-eight blood donors. Tests were run on the automated slide processor ZENIT FLOW, and then the slides were imaged and analyzed using ZENIT fast. Results: Since the samples were selected for at least one antibody positivity with an ARD diagnosis, we did not calculate clinical sensitivity but only specificity, which was 98.53%, ranging from 90% for anti-SSB/La antibodies to 100% for anti-CENP B ones. Mean agreement among the methods assessed by Cohen’s kappa was 0.816 ± 0.240. Conclusions: The assay demonstrated good clinical performance and may be considered a valuable aid in detecting ARD patients, offering an alternative to methods such as FEIA which are largely in use today.

## 1. Introduction

In recent years, the panorama of autoimmune disease diagnostics has been affected by huge changes regarding technical–analytical and instrumental innovations, which have brought about new solutions to critical situations, but they have also required professionals and structures to make great effort in managing the organization of immunology laboratories [1,2,3]. Increasing knowledge about autoimmune rheumatic diseases (ARDs) has led to a more accurate understanding of the clinical significance of many autoantibodies and the need to introduce new instrumental platforms for the measurement of circulating antibodies [4,5,6,7,8,9].

Due to its high sensitivity, the use of anti-nuclear antibodies (ANAs) by indirect immunofluorescence (IFI) on HEp-2 cells is common for the screening of autoantibodies, although the visual evaluation is time-consuming, subjective, and it requires trained personnel [10,11,12].

In addition to providing information on the presence of several autoantibodies, the HEp- 2 IFI also provides valuable information on the serum levels (given by the titer of reactivity) [13] and the possible specificity of the autoantibodies in the sample (given by the immunofluorescence pattern) [14]. For these reasons, the search for ANAs in IFI on HEp2 cells has all the characteristics for it to be used as a first level test compared to monoplex and multiplex immunometric tests, which are more specific and more expensive and require complex instrumentation for the identification and quantification of individual specificities. In particular, the methods for measuring the specificities of extractable nuclear antigens (ENAs) have changed profoundly over time, moving from the first singleplex analytical methods to multiplex platforms, which have allowed for the simultaneous detection of an increasingly large panel of autoantibodies [15]. Initially, the application of first-generation immunochemical methods such as passive hemagglutination, double immunodiffusion, counterimmunoelectrophoresis, radioimmunoprecipitation, and Western blot [16,17] allowed for qualitative and semiquantitative analyses to be conducted; however, the identification of new antigenic molecules and understanding their clinical significance have required the introduction of new analytical and instrumental platforms for a broader autoantibody panel [18].

Compared to a few years ago, the modern immunology laboratory has many new validated techniques to detect antibodies, such as line immunoassay (LIA), chemiluminescence immunoassay (CLIA), fluoroimmunoenzyme immunoassay (FEIA) and microarray, which allow for the study of a more expanded autoantibodies panel [19,20,21,22,23,24,25,26].

In the present paper, we have evaluated a new slide microarray test, the Zenit AmiDot CTD panel, to determine anti-CENP B and anti-extractable nuclear antigen (ENA) specificity, including anti-Ro60, anti-Ro52, anti-SSB/La, anti-U1-RNP, anti-Scl-70, and anti-Jo-1.

We have compared the performance of the Zenit AmiDot CTD panel with the EliA test, an FEIA method commonly used in current laboratory practice to detect anti-ENA antibodies.

## 2. Materials and Methods

### 2.1. Patients

Sixty-nine consecutive serum samples from ARD patients attending the Immunology and Allergology laboratory of San Giovanni di Dio Hospital (Florence) were collected to test for anti-ENA antibodies. Sixty-eight blood donors’ sera were included as the control group in the study, with an F:M ratio of 4.1 and mean age = 62.9 ± 15.9 (SD), matched by sex and age. ARD diagnoses were determined according to internationally validated disease criteria.

The distribution of the different ARDs is as follows: 24 systemic sclerosis (SSc), 21 Sjögren’s syndrome (SjS), 16 systemic lupus erythematosus (SLE), and 8 polymyositis/dermatomyositis (PM/DM) (Table 1).

### 2.2. Methods

The ZENIT AmiDot CTD panel was performed on clinical samples, and the results were compared to those obtained by the FEIA EliA method for the detection of anti-ENA antibodies. The discordant results were further evaluated by two other different methods: a CLIA method (ZENIT PRIME, A. Menarini Diagnostics, Florence, Italy) and an LIA method (Euroline ANA profile3 plus DFS70 IgG, Euroimmun, Luebeck, Germany).

#### 2.2.1. ZENIT AMiDot CTD panel

The ZENIT AMiDot CTD panel (A. Menarini Diagnostic, Florence, Italy) is a new fluorescence microarray test used for the detection, identification, and in vitro quantification of antibodies anti-Ro60, -Ro52, -SSB/La, -Sm, -U1snRNP (A/C/68kD), -dsDNA, -nucleosome, -Jo-1, -Sc170, -PMScl100, -ribosomal P protein P0, -CENP B, -PCNA, -Mi-2, -Ku, -DFS70, -PL7/PL12, -M2, -Sp100, and -gp210 in human serum or plasma samples.

**Figure 1:**
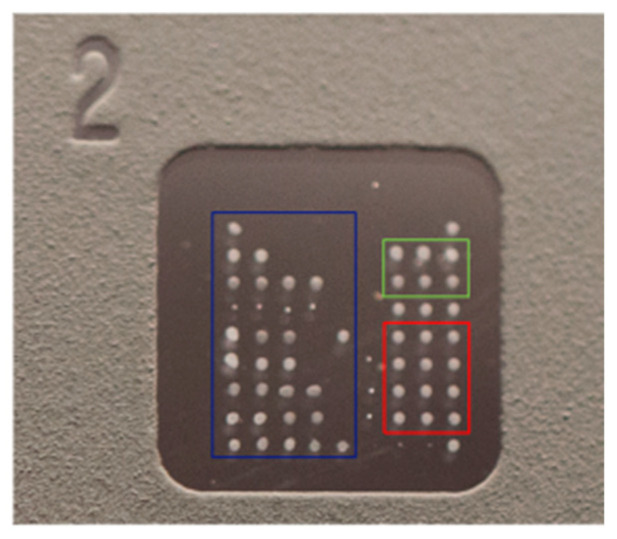
ZENIT AMiDot single well.

The slide consists of eight microarray wells with immobilized antigens. Each well contains a matrix of 9 × 9 dots: 60 (20 × 3) dots constitute triplicates of each immobilized antigen, six wells make up the calibrator triplicates (0 ng/mL and 25 ng/mL), 6 dots constitute the triplicates of the positive control (anti-human IgG) and conjugate control, and 4 dots constitute the registration dots, i.e., an internal control used for visual inspection during the production process to verify that the slide is correctly printed (Figure 1 and Table 2).

Antibodies are detected using an FITC-conjugated goat anti-human IgG.

The ZENIT AMiDot CTD panel can be processed both manually and automatically. In this study, the ZENIT FLOW automatic system (A. Menarini Diagnostics) was used. ZENIT FLOW is an automated device used to process indirect immunofluorescence test kits, starting from sample dilutions (serum, plasma) all the way up to covering the slide with a coverslip.

The ZENIT FLOW system includes a robotic liquid handling unit designed for slide processing and specific management software. The liquid handling system aspirates and dispenses samples, reagents, controls, diluted samples, mounting medium, and three-needle wash solutions in combination with peristaltic and volumetric pumps (syringes). After diluting the samples, the system dispenses the PBS-conjugated antibody and washes and adds the reagents sequentially to the wells of each slide. ZENIT FLOW is able to automate the preparation of 30 slides in a single session by planning each phase to guarantee uniform incubation times on each slide. After the incubation and washing procedure, the slide is automatically mounted; the mounting medium is dispensed onto the slide, and a dedicated suction cap located in the robotic arm positions the coverslip to cover and seal the slide, thereby avoiding the formation of bubbles.

The ZENIT FLOW, through a specific protocol, is able to automate all the steps necessary to perform the ZENIT AMiDot CTD panel assay.

Before proceeding with the test, all the reagents are brought to room temperature (18–25 °C) for at least 2 h prior to use. Subsequently, all the samples and reagents are loaded onto the ZENIT FLOW instrument in the appropriate racks of the analysis session. Each sample is diluted 1:80 (10 µL of sample + 790 µL of sample diluent), dispensed into the well (60 µL), and left to incubate for 30 min. In this phase, the autoantibodies present in the patient’s serum bind to the antigen or antigens immobilized on the microarray. Once the incubation time has elapsed, the slide undergoes a first washing step with five drops (50 µL) of wash buffer to remove antibodies that did not recognize the specific antigen.

The bound autoantibodies are detected by dispensing onto the well 50 µL of a goat anti-human IgG antibody conjugated to an FITC fluorescent molecule. After 30 min of incubation with the conjugated antibody, the slide is washed again with five drops (50 µL) of wash buffer. Subsequently the whipping liquid is added, and the slide is covered with a coverslip to be read with the ZENIT fast fluorescence microscope (A. Menarini Diagnostics).

ZENIT fast is an automatic system that consists of a fluorescence microscope with a LED light source, a motorized stage, a CCD camera, a computer, a keyboard and a mouse, a monitor, and specific software for the analysis.

Thanks to a specific module for reading the ZENIT AmiDot CTD panel, the ZENIT fast reader software measures the average fluorescence intensity of each antigen distributed in triplicate and calculates its concentration based on the calibration curve. The cut-off value is 10 AU/mL.

#### 2.2.2. FEIA method (EliA Test) for Anti-ENA specificities

The EliA test is a fluorescence enzyme immunoassay (FEIA) performed using Unicap 250 instrumentation (Thermo Fisher, Uppsala, Sweden) to identify anti-ENA antibodies and other autoantibodies.

The EliA assay involves the use of wells coated with one or more target antigens, which are recognized and bound by specific autoantibodies. If the patient sample contains the specific antibodies, they bind to the corresponding target antigen in the EliA well. After washing away non-bound antibodies, enzyme-labeled antibodies against human IgG antibodies (EliA IgG Conjugate) are added to form an antibody–conjugate complex. After incubation, the non-bound conjugate is washed away, and the bound complex is incubated with a development solution. Antibodies bind specifically to the Fc region of the patient’s IgG antibodies. After a second washing step, in which excess secondary antibodies are removed, a reagent is added to the antigen–antibody complex. This reagent is converted into a fluorescent substrate through an enzymatic reaction. After an appropriate incubation time, the enzymatic reaction is stopped by using a stop solution, and the fluorescence is measured with a fluorometer.

The concentration of antibodies in the patient sample is determined using the standardized calibration curve previously set. The resulting quantitative result allows for the classification of the sample as negative, borderline, or positive.

The cut-off value is 10 AU/mL for all antibodies.

#### 2.2.3. CLIA PRIME ZENIT

CLIA ZENIT PRIME is a fully automated immunoanalyzer, provided by A. Menarini Diagnostics, in which concentrations are expressed as arbitrary AU/mL. The cut-off value used was 10 AU/mL. The assay uses a two-step immunoassay method based on the principle of chemiluminescence, using antigen-coated magnetic particles as the solid phase and an antibody labeled with a dimethyl acridinium ester as the detection marker.

Antibodies to the main ENA specificities were detected using seven different kits, each containing the specific antigen attached to the solid phase microspheres. In particular, Sm is a native antigen, while Ro60, Ro52, SSB/La, Scl70, Jo1, U1-RNP, and CENP B are recombinant. 

#### 2.2.4. LIA Euroline ANA Profile 3 plus DFS70 IgG

The LIA Euroline ANA Profile 3 plus DFS70 IgG is a line blot that involves the use of strips containing the following antigens: RNP/Sm, Sm, SS-A, Ro-52, SS-B, Scl-70, PM-Scl, Jo-1, DFS-70, CENP B, ribosomal protein, dsDNA, nucleosomes, histones, and the AMA-M2 component. The test kit contains test strips coated with parallel lines of highly purified antigens. In the first reaction step, diluted patient samples are incubated with the immunoblot strips. In the case of positive samples, the specific IgG antibodies will bind to the corresponding antigenic site. To detect the bound antibodies, a second incubation is carried out using an enzyme-labeled anti-human IgG (enzyme conjugate) catalyzing a color reaction. After stopping the reaction using deionized or distilled water, the incubated test strips are placed onto the adhesive foil of the green work protocol using a pair of tweezers. After they have dried, the test strips are stuck to the adhesive foil. The dry test strips are then scanned using a flatbed scanner and evaluated with EUROLineScan. For the LIA assay, semiquantitative results were obtained, considering values greater than 15 AU as positive. 

## 3. Statistical Analysis

The concordance between positive and negative results obtained through the two methods used (FEIA and AMiDot) was assessed by Cohen’s k test. The percentage of concordance between the two tests, obtained from the ratio between the number of concordances (positive or negative) and the total number of cases evaluated, was also calculated. The analyses were conducted with the statistical software SPSS (IBM Armonk, New York, NY, USA, v28). A value of *p* < 0.05 was considered statistically significant.

## 4. Results

In this study, 69 samples from patients diagnosed with ARDs were analyzed with two different methods (AMiDot and FEIA) for the detection of anti-CENP B and six anti-ENAs: anti-Scl-70, anti-SSB/La, anti-Jo-, anti-U1-RNP, anti-Ro52, and anti-Ro60 antibodies.

**Figure 2:**
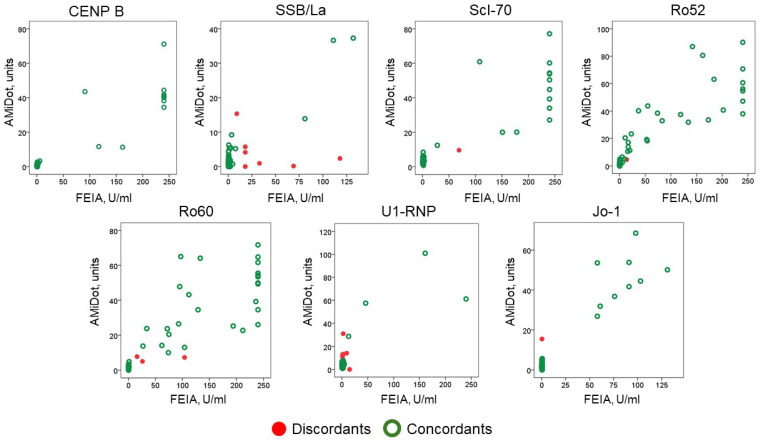
Distribution of anti-CENP B and anti-ENA antibodies results (concordants and discordants) by the two methods, AMiDot and FEIA.

The distribution of values across the scatter plot is represented in Figure 2.

Since the ARD patients were selected for at least one antibody positivity, we did not evaluate the clinical sensitivity but rather the specificity, which was 98.53%.

The agreement by Cohen’s kappa showed a mean of 0.816 ± 0.240 with a minimum value of 0.415 for anti-SSB/La antibodies and a maximum value of 1.000 for anti-CENP B (Table 3).

There were 19 discordant samples: 12 FEIA+/AMiDot− and 7 AMiDot+/FEIA− (Table 3).

For the discordant samples, two additional methods, CLIA (PRIME ZENIT) and LIA (Euroline ANA Profile 3 plus DFS70 IgG), were further used (Table 4).

Out of the FEIA+/AMiDot− samples, five were double positive (FEIA+/PRIME ZENIT+ or FEIA+/LIA+) and five were triple positive (FEIA+/PRIME ZENIT+/LIA+).

Of the AMiDot+/FEIA− samples, one was double positive (AMiDot+/LIA+) and one was triple positive (AMiDot+/PRIME ZENIT+/LIA+).

Regarding the discordance of the anti-U1-RNP antigen, we could not consider the LIA method, as it does not include it in the panel.

Of the six discordant samples for this antigen, the FEIA+/AMiDot− sample was positive by the third PRIME ZENIT CLIA method. Of the five AMiDot+/FEIA− samples, three were positive by the PRIME ZENIT CLIA method.

For a better understanding of the discrepancies, the nature of the antigen in the tests under study is reported in Table 5. All antigens were recombinant, except for Scl-70 (LIA and AMiDot), Jo1, Ro60, and SSB/La (LIA).

## 5. Discussion

Since the presence of anti-ENA antibodies represents a specific ARD marker and is often listed for disease classification or as a diagnostic criterion, the method choice for their detection is a topic of great interest and relevance today.

The methods currently used in clinical laboratories to determine anti-ENA antibodies are continuously increasing, and consequently harmonizing the tests is growing more difficult.

Furthermore, due to the absence of international standards on the market, the standardization of antibody assays is still a very long road, hindered by the significant differences in results from the various tests used [27]. Analytical variability is linked not only to the method used, but also to the kind of binding to solid supports and to the nature of the antigen (recombinant or extractive).

Numerous comparative studies exist in the literature on anti-ENA antibodies assays, but in most cases, they are limited to a few methods, to a specific antibody, or to a specific ARD cohort [23,26].

Recently, a multicenter study conducted by the Autoimmune Diseases Study Group of the Italian Society of Laboratory Medicine (SIPMeL) evaluated a population of 60 patients with a diagnosis of ARD and 20 subjects as control group for anti-ENA antibodies by eight different assays (dot blot, CLIA, addressable laser bead immunoassays, FEIA, microarray, LIA, enzyme linked immunoassay), with an average of inter-assays agreement of 82%. Although specificity was excellent for all methods, some differences were highlighted regarding analytical sensitivity.

In the present study, we evaluated the diagnostic performance of an innovative slide microarray test introduced on the market, the ZENIT AMiDot CTD panel, for the detection of anti-ENAs, also comparing it to an FEIA method widely used in clinical laboratories since, to date, there is no gold standard reference test. The diagnostic performance of the test was very good, and the comparison with the FEIA method showed a different percentage of κ agreement based on the specific antibody considered from 0.415 (anti-SSB/La) to 1.000 (anti-CENP B) with an average of 0.816, in line with the multicenter study recently carried out by the SIPMeL Autoimmune Diseases Study Group [25]. 

In 2016, Dillaerts et al. performed the AMiDot on 184 samples from blood donors and on 280 randomly selected clinical samples containing antibodies to ENAs. The κ agreement between AMiDot and FEIA was ≥0.44 (≥0.70 after exclusion of anti-SmD) [28]. The low agreement of anti-Sm was related to the fact that AMiDot uses native Sm as the antigen, but FEIA EliA uses the synthetic antigen SmD. For this reason, we decided to exclude the comparison of anti-Sm antibodies from the design of our study. However, even if the authors considered two different cutoffs for the AMiDot, the percentage of concordance obtained was in line with our study for the majority of the anti-ENA antibodies.

Moreover, compared to Dillaerts et al.’s study, the New AmiDot CTD panel test evaluated in this case is a new generation test. Test performance has been improved by a new coating process that ensures a more stable covalent binding of the antigens to the glass. The new coating also made it possible to remove the activation buffer included in the old version of the kit which was used to make the antigen binding sites more accessible.

In addition to this, the new AmiDot CTD panel was automated on the ZENIT FLOW processor, whose coverslipping step allowed for the elimination of the washing step which was necessary in the older version to avoid salt residues that could create interference. Compared to manual processing, ZENIT FLOW enables better image focusing, including with regard to dust residues.

Recently, Norimatsu et al. tested a novel autoantibody array assay, A-cube, showing comparable performances with existing tests and, in addition, the possibility to detect anti-OJ antibodies that could not be detected using conventional tests [29].

One limitation of our study is the small number of samples tested, which is why we did not carry out a stratified analysis by the different antibodies or pathology. Furthermore, we used a control group of donors but not a disease control group, which could have improved the evaluation of the specificity of the antibodies test.

Another limitation is the anti-ENA antibodies panel studied, which did not include some minor specificities of the ANAs, such as anti-fibrillarin, -Ku, and -RiboP, present in the AMiDot CTD panel but of a rare diagnostic finding; for this reason, we did not take them into consideration in our study.

In our study, the AMiDot CTD panel has shown a good clinical performance and therefore may represent a valid alternative to the current commercially available tests, offering some interesting advantages such as the use of a single platform for AMiDot and immunofluorescence tests (same slides, LEDs, and filter for reading) and different antigen panel readings that can be qualitative and quantitative. Hence, this enables laboratories to perform in parallel both screening and specificity autoantibodies’ confirmation, enhancing the efficiency of autoimmune diagnosis; the great advantage is that very small volumes of serum samples (10 uL) are required to detect multiple specificities. Compared to CLIA, FEIA, and other anti-ENA immunoassay techniques, AMiDot controls and calibrators are coated in each single well, limiting additional assay procedures and costs.

In-depth knowledge of the methods and awareness of their analytical variability are of fundamental importance, on the one hand, for the laboratory personnel to choose the “best method”, based on their own clinical context and internally adopted work algorithms, and on the other, for clinical specialists to correctly and appropriately interpret the laboratory findings report.

## Figures and Tables

**Table 1:** Demographic characteristics of patients.

ARD	Patients (n)	Ratio F/M	Age (Years) Mean ± SD	Disease Duration (Days) Mean ± SD
SSc	24	17/7	62.0 ± 12.0	85.6 ± 73.5
SjS	21	15/6	69.2 ± 9.7	182.4 ± 13.1
SLE	16	10/6	58.4 ± 13.5	122.4 ± 61.6
PM/DM	8	7/1	60.3 ± 13.7	145.4 ± 43.1

SSc: systemic sclerosis; SjS: Sjögren’s syndrome; SLE: systemic lupus erythematosus; PM/DM: polymyositis/dermatomyositis.

**Table 2:** ZENIT AMiDot CTD panel slide layout.

	1	2	3	4	5	6	7	8	9
1	Registration	0 ug/mL Cal	0 ug/mL Cal	0 ug/mL Cal	Positive Control	Positive Control	Positive Control		Registration
2		25 ug/mL Cal	25 ug/mL Cal	25 ug/mL Cal	Conjugate Control	Conjugate Control	Conjugate Control		
3	Ro60	Ro52	SSB/La	Sm	RNP A/C/68kD	Jo-1	Scl70	PMScl100	dsDNA
4	Ro60	Ro52	SSB/La	Sm	RNP A/C/68kD	Jo-1	Scl70	PMScl100	dsDNA
5	Ro60	Ro52	SSB/La	Sm	RNP A/C/68kD	Jo-1	Scl70	PMScl100	dsDNA
6	Ribosomal P0	CENP B	DFS70	PCNA	Ku	Mi-2	PL7/PL12	M2	Nucleosome
7	Ribosomal P0	CENP B	DFS70	PCNA	Ku	Mi-2	PL7/PL12	M2	Nucleosome
8	Ribosomal P0	CENP B	DFS70	PCNA	Ku	Mi-2	PL7/PL12	M2	Nucleosome
9	Registration	sp100	sp100	sp100		gp210	gp210	gp210	Registration

**Table 3:** Agreement by Cohen’s kappa between autoantibodies tested by AMiDot and FEIA methods.

	k	k (95% CI)	*p*-Value	AMiDot(+) FEIA(+)	AMiDot(−) FEIA(−)	AmiDot(+) FEIA(−)	AmiDot(−) FEIA(+)	Concordance (%) AMiDot-FEIA
CENP B	1.000	1.000–1.000	<0.001	10	59	0	0	100%
SSB/La	0.415	0.070–0.759	<0.001	3	59	1	6	90%
Scl-70	0.954	0.865–1.000	<0.001	13	55	0	1	99%
Ro52	0.969	0.910–1.000	<0.001	26	42	0	1	99%
Ro60	0.911	0.812–1.000	<0.001	27	39	0	3	96%
U1-RNP	0.527	0.201–0.854	<0.001	4	59	5	1	91%
Jo-1	0.939	0.821–1.000	<0.001	9	59	1	0	99%

Mean k ± standard deviation: 0.816 ± 0.240 (95% CI: 0.6–1.0). CI: confidence interval.

**Table 4:** List of discordant sera results between AMiDot and FEIA methods: additional evaluation by the alternative CLIA and LIA methods.

SAMPLE	ID SAMPLE	DISCORDANT ANTIBODY	FEIA (U/mL) TermoFisher	AMiDot (AU/mL) A. Menarini Diagnostics	ZENIT PRIME (AU/mL) A. Menarini Diagnostics	LIA (AU) Euroimmun	CLINICAL DIAGNOSIS
1	4340714165	Scl-70	POS (69)	NEG (9.63)	POS (121.7)	POS (53)	SSc
2	4340714165	Jo-1	NEG (0.4)	POS (15.48)	POS (49.5)	POS (160)	SSc
3	4340122320	SSB/La	POS (33)	NEG (0.93)	POS (16.3)	POS (59)	SjS
4	4340125659	SSB/La	POS (118)	NEG (2.36)	POS (82.24)	POS (51)	SjS
5	4340125661	SSB/La	POS (18)	NEG (5.74)	NEG (7.6)	POS (19)	SjS
6	4340125688	SSB/La	NEG (9.1)	POS (15.34)	NEG (1.9)	POS (27)	SjS
7	4340125761	SSB/La	POS (69)	NEG (0.14)	POS (24)	POS (31)	SjS
8	4340126222	SSB/La	POS (18)	NEG (<0.01)	NEG (0)	POS (19)	SjS
9	4345098797	SSB/La	POS (18)	NEG (4.13)	NEG (8.6)	POS (19)	SjS
10	4340126647	Ro60	POS (16)	NEG (7.74)	POS (20.3)	POS (46)	SSc
11	4340127827	Ro60	POS (104)	NEG (7.27)	NEG (6.7)	POS (65)	SLE
12	4340083266	Ro60	POS (26)	NEG (5.03)	Insufficient Sample	NEG (14)	SSc
13	4340126585	Ro52	POS (14)	NEG (4.63)	NEG (4.4)	POS (91)	SjS
14	4340126638	U1-RNP	POS (15)	NEG (<0.01)	POS (14.4)	nd	SLE
15	4340718515	U1-RNP	NEG (2.6)	POS (31.01)	POS (31.4)	nd	SSc
16	4340715372	U1-RNP	NEG (1.8)	POS (13.22)	POS (27.6)	nd	SSc
17	4340083266	U1-RNP	NEG (1.4)	POS (12.43)	NEG (1.4)	nd	SSc
18	4327002104	U1-RNP	NEG (9.2)	POS (14.08)	POS (18.9)	nd	PM/DM
19	4340095147	U1-RNP	NEG (1.2)	POS (10.98)	NEG (5.9)	nd	PM/DM

nd: not determinable, POS: positive, NEG: negative. Cut-off FEIA: >10 AU mL positive. Cut-off AMiDot: >10 AU/mL positive. Cut-off CLIA: >10 AU/mL positive. Cut-off LIA: >15 AU positive.

**Table 5:** Characteristics of anti-ENA antibodies in the ZENIT AMiDot, LIA, FEIA, and CLIA methods.

	ANTIGEN							
ASSAY (METHODS)	MANUFACTURER	Scl-70	CENP B	Jo-1	Ro60	Ro52	SSB/La	U1-RNP
**AMiDot**	A. Menarini Diagnostics	N	R	R	R	R	R	R
**LIA**	Euroimmun	N	R	N	N	R	N	na
**FEIA**	TermoFisher Scientific	R	R	R	R	R	R	R
**CLIA**	A. Menarini Diagnostics	R	R	R	R	R	R	R

N: native/extractive; R: recombinant; na: not applicable.

## Data Availability

The original contributions presented in the study are included in the article; further inquiries can be directed to the corresponding author/s.

## References

[1] Tozzoli R. (2007). Recent advances in diagnostic technologies and their impact in autoimmune diseases. Autoimmun. Rev..

[2] Sack U., Bossuyt X., Andreeva H., Antal-Szalmás P., Bizzaro N., Bogdanos D., Borzova E., Conrad K., Dragon-Durey M.-A., Eriksson C. (2020). European Autoimmunity Standardisation Initiative. Quality and best practice in medical laboratories: Specific requests for autoimmunity testing. Auto. Immun. Highlights.

[3] Sciascia S., Bizzaro N., Meroni P.L., Dimitrios B., Borghi M.O., Bossuyt X., Grossi C., Tornai D., Papp M., Shoenfeld Y. (2023). Autoantibodies testing in autoimmunity: Diagnostic, prognostic and classification value. Autoimmun. Rev..

[4] Tozzoli R., D’Aurizio F., Villalta D., Bizzaro N. (2015). Automation, consolidation, and integration in autoimmune diagnostics. Auto. Immun. Highlights.

[5] Lalvani A., Meroni P.L., Millington K.A., Modolo M.L., Plebani M., Tincani A., Villalta D., Doria A., Ghirardello A. (2008). Recent advances in diagnostic technology: Applications in autoimmune and infectious diseases. Clin. Exp. Rheumatol..

[6] Sharp V., Utz P.J. (2007). Technology insight: Can autoantibody profiling improve clinical practice?. Nat. Clin. Pract. Rheumatol..

[7] Plebani M., Pittoni M., Celadin M., Bernardi D., Mion M.M. (2009). Recent advances in diagnostic technologies for autoimmune diseases. Autoimmun. Rev..

[8] Cinquanta L., Infantino M., Bizzaro N. (2022). Detecting Autoantibodies by Multiparametric Assays: Impact on Prevention, Diagnosis, Monitoring, and Personalized Therapy in Autoimmune Diseases. J. Appl. Lab. Med..

[9] Bizzaro N. (2019). Autoantibody Profiles in Autoimmune Rheumatic Diseases. Mediterr. J. Rheumatol..

[10] Bossuyt X., De Langhe E., Borghi M.O., Meroni P.L. (2020). Understanding and interpreting antinuclear antibody tests in systemic rheumatic diseases. Nat. Rev. Rheumatol..

[11] Bonroy C., Vercammen M., Fierz W., Andrade L.E.C., Van Hoovels L., Infantino M., Fritzler M.J., Bogdanos D., Kozmar A., Nespola B. (2023). Detection of antinuclear antibodies: Recommendations from EFLM, EASI and ICAP. Clin. Chem. Lab. Med..

[12] Meroni P.L., Schur P.H. (2010). ANA screening: An old test with new recommendations. Ann. Rheum. Dis..

[13] Vulsteke J.B., Van Hoovels L., Willems P., Vander Cruyssen B., Vanderschueren S., Westhovens R., Blockmans D., De Langhe E., Bossuyt X. (2021). Titre specific positive predictive value of anti-nuclear antibody patterns. Ann. Rheum. Dis..

[14] Damoiseaux J., Andrade L.E.C., Carballo O.G., Conrad K., Francescantonio P.L.C., Fritzler M.J., de la Torre I.G., Herold M., Klotz W., Cruvinel W.d.M. (2019). Clinical relevance of HEp-2 indirect immunofluorescent patterns: The International Consensus on ANA patterns (ICAP) perspective. Ann. Rheum. Dis..

[15] Tozzoli R., Bonaguri C., Melegari A., Antico A., Bassetti D., Bizzaro N. (2013). Current state of diagnostic technologies in the autoimmunology laboratory. Clin. Chem. Lab. Med..

[16] Nakamura R.M., Tan E.M. (1978). Recent progress in the study of autoantibodies to nuclear antigens. Hum. Pathol..

[17] De Rooij D.J., van de Putte L.B., Habets W.J., Verbeek A.L., van Venrooij W.J. (1988). The use of immunoblotting to detect antibodies to nuclear and cytoplasmic antigens. Clinical and serological associations in rheumatic diseases. Scand. J. Rheumatol..

[18] Tozzoli R., Villalta D., Bizzaro N. (2017). Challenges in the Standardization of Autoantibody Testing: A Comprehensive Review. Clin. Rev. Allergy Immunol..

[19] Robinson W.H., DiGennaro C., Hueber W., Haab B.B., Kamachi M., Dean E.J., Fournel S., Fong D., Genovese M.C., De Vegvar H.E.N. (2002). Autoantigen microarrays for multiplex characterization of autoantibody responses. Nat. Med..

[20] Carbone T., Infantino M., Antico A., Porcelli B., Villalta D., Pafundi V., Bizzaro N. (2023). An Italian nationwide survey on the evolution of autoantibody diagnostics in autoimmune rheumatic diseases. Clin. Exp. Rheumatol..

[21] González D.A., de León A.C., Pérez M.D.C.R., Díaz B.B., Hernández A.G., García D.G., Moncholi C.V., Jaime A.A. (2010). Efficiency of different strategies to detect autoantibodies to extractable nuclear antigens. J. Immunol. Methods.

[22] Lee S.A., Kahng J., Kim Y., Park Y.J., Han K., Kwok S.K., Park S.H., Oh E.J. (2012). Comparative study of immunofluorescent antinuclear antibody test and line immunoassay detecting 15 specific autoantibodies in patients with systemic rheumatic disease. J. Clin. Lab. Anal..

[23] López-Longo F.J., Rodríguez-Mahou M., Escalona-Monge M., González C.M., Monteagudo I., Carreño-Pérez L. (2003). Simultaneous identification of various antinuclear antibodies using an automated multiparameter line immunoassay system. Lupus.

[24] Mahler M., Betteridge Z., Bentow C., Richards M., Seaman A., Chinoy H., McHugh N. (2019). Comparison of Three Immunoassays for the Detection of Myositis Specific Antibodies. Front. Immunol..

[25] Infantino M., Carbone T., Brusca I., Alessio M.G., Previtali G., Platzgummer S., Paura G., Castiglione C., Fabris M., Pesce G. (2022). Study Group on Autoimmune Diseases of the Italian Society of Clinical Pathology and Laboratory Medicine. Current technologies for anti-ENA antibody detection: State-of-the-art of diagnostic immunoassays. J. Immunol. Methods.

[26] Infantino M., Bentow C., Seaman A., Benucci M., Atzeni F., Sarzi-Puttini P., Olivito B., Meacci F., Manfredi M., Mahler M. (2013). Highlights on novel technologies for the detection of antibodies to Ro60, Ro52, and SS-B. Clin. Dev. Immunol..

[27] Chan E.K., Fritzler M.J., Wiik A., Andrade L.E., Reeves W.H., Tincani A., Meroni P.L., IUIS/WHO/AF/CDC Committee for the Standardization of Autoantibodies in Rheumatic and Related Diseases (2007). AutoAbSC.Org—Autoantibody Standardization Committee in 2006. Autoimmun. Rev..

[28] Dillaerts D., De Baere H., Bossuyt X. (2017). Clinical autoantibody detection by microarray. Clin. Chem. Lab. Med..

[29] Norimatsu Y., Matsuda K.M., Yamaguchi K., Ono C., Okumura T., Kogo E., Kotani H., Hisamoto T., Kuzumi A., Fukasawa T. (2023). The Autoantibody Array Assay: A Novel Autoantibody Detection Method. Diagnostics.

